# Soliton dynamics in the stochastic nonlinear Schrödinger equation with self-phase modulation and multiplicative white noise

**DOI:** 10.1038/s41598-026-53450-2

**Published:** 2026-05-27

**Authors:** Mohammed F. Shehab, Hamdy M. Ahmed, Hisham H. Hussein

**Affiliations:** 1 Department of Basic Sciences, Faculty of Engineering Technology, ElSewedy University of Technology, Cairo, Egypt; 2 Department of Physics and Engineering Mathematics, Higher Institute of Engineering, El-Shorouk Academy, El-Shorouk city, Cairo Egypt; 3 School of Mathematical and Computational Sciences, University of Prince Edward Island (UPEI), Cairo Campus, The New Administrative Capital, Egypt

**Keywords:** Soliton solutions, Stochastic nonlinear Schrödinger equation, Wiener process, Improved modified extended tanh-function method., Mathematics and computing, Optics and photonics, Physics

## Abstract

In this study, we investigate the stochastic nonlinear Schrödinger equation incorporating self-phase modulation under the influence of multiplicative white noise in the dispersionless regime. By employing the improved modified extended tanh function method , we derive a rich spectrum of analytical solutions, including bright and dark solitons, singular and periodic structures, as well as solutions represented through Jacobi and Weierstrass elliptic functions. This analytical framework not only provides a systematic approach for capturing accurate solutions in noisy environments but also provides an effective analytical approach in addressing nonlinear stochastic partial differential equations. We present a thorough graphical analysis that shows solution behavior across various noise intensity regimes and methodically examine the effects of stochastic perturbations on soliton propagation dynamics. The proposed approach provides an analytical framework for constructing exact wave solutions to the considered model and demonstrates its applicability through several representative solution structures.

## Introduction

A basic model in mathematical physics, the stochastic nonlinear Schrödinger equation (SNLSE) extends the standard nonlinear Schrödinger equation by incorporating noise effects and random fluctuations^1,2^. The growing importance of stochastic extensions for explaining wave propagation in physical systems where external noise is significant has motivated considerable research in this area. In this broader context, related stochastic evolution equations, such as the stochastic Burgers equation, have also been investigated using analytical and numerical methods to better understand the role of noise in nonlinear systems^3^. In modeling wave dynamics in optical fiber communications and other nonlinear media, stochastic effects play a central role in shaping the qualitative behavior of solutions. Building on recent advances in the mathematical theory of stochastic nonlinear Schrödinger equations, these models have proven valuable for applications ranging from optical communication systems to ecological settings under environmental noise^4^. The three-component nonlinear stochastic Schrödinger equation, for example, has been used to model pulse propagation in birefringent optical fibers subject to Brownian-driven Stratonovich noise, where diverse solution families such as dark, bright, singular, combined, and solitary optical solitons can be obtained, together with descriptions of how their profiles are deformed by the noise^5^. The extended Klausmeier-type stochastic framework similarly demonstrates how environmental noise reshapes solitary and periodic wave patterns, emphasizing the broader role of stochastic perturbations in modifying soliton structures in nonlinear dispersive systems^6^. Understanding how noise affects wave propagation remains challenging, because stochastic perturbations can significantly change the qualitative behavior of solutions and generate new emergent features. Recent studies on perturbed and stochastic nonlinear Schrödinger equations show that Wiener-driven noise and higher-order effects can produce diverse soliton families and stability regimes, offering both methodological guidance and physical motivation for studying stochastic nonlinear wave models^7^.

The phenomenon of self-phase modulation (SPM) in optical systems represents one of the most important nonlinear effects in wave propagation, particularly in the absence of chromatic dispersion^8,9^. Ultrashort pulse propagation in optical media leads to a rapid modulation of the refractive index through the Kerr nonlinearity. This modulation induces a temporal evolution of the optical phase along the pulse, giving rise to self-phase modulation through the intrinsic coupling between the electromagnetic field and the material response. This phenomenon is important for understanding nonlinear dynamics in photonics applications, particularly in optical fibers and laser systems, because it produces a time-dependent phase shift of optical pulses as they propagate through different media. When chromatic dispersion effects are negligible or absent, SPM becomes the dominant nonlinear mechanism, leading to spectral broadening and temporal pulse evolution that can be modeled through appropriately formulated nonlinear Schrödinger equations^8^. For this reason, the present model was chosen to study the interaction between stochastic perturbations and self-phase modulation in the dispersionless regime, where the role of SPM can be examined more directly.

Multiplicative white noise fundamentally alters nonlinear Schrödinger equation behavior by introducing amplitude-dependent stochastic fluctuations that scale directly with the wave function magnitude, creating interactions that are absent in additive noise scenarios [1,2]. Unlike additive noise, which uniformly affects all components, multiplicative white noise exhibits amplitude-dependent characteristics, where larger amplitude regions experience proportionally stronger perturbations, leading to enhanced instabilities and modified soliton dynamics^10^. This creates a feedback mechanism in which stochastic perturbations are inherently coupled to deterministic evolution, resulting in phenomena such as noise-induced transitions and changes in qualitative dynamical properties that distinguish these systems from their deterministic counterparts^11^. Therefore, the inclusion of multiplicative white noise in the present model is physically relevant and mathematically meaningful for describing nonlinear wave propagation in randomly perturbed optical media.

Various mathematical techniques have been explored for constructing solutions to nonlinear partial differential equations (NPDEs), ranging from classical analytical methods to modern computational approaches. Among these, the Bilinear Neural Network Method and its improved variant have emerged as efficient methodologies that integrate machine learning to extract complex solutions such as rogue waves and lumps in systems including graphene sheets and shallow water models^12,13^. Concurrently, the classical Hirota Bilinear Transformation continues to serve as a fundamental technique for systematically deriving exact solutions, including anti-kink waves and soliton crystals, in nonlinear frameworks such as nonlinear atom chains and the Chaffee-Infante model^14,15^. To tackle the mathematical challenges posed by the stochastic nonlinear Schrödinger equation with SPM effects, advanced analytical techniques are needed to obtain exact solutions that provide physical insight into the underlying wave dynamics^16–19^.

In this regard, the improved modified extended tanh function method (IMETF) represents a useful analytical tool for solving nonlinear evolution equations^20–24^. By employing an ansatz that converts complex partial differential equations into manageable algebraic systems, the IMETF method allows the derivation of several exact solution forms within a unified framework. Compared with previous related studies, the present work focuses on a more specific physical setting, namely the stochastic nonlinear Schrödinger equation with self-phase modulation under multiplicative white noise in the dispersionless regime. This choice enables a more direct examination of the interplay between SPM and amplitude-dependent stochastic perturbations. In addition, the present analysis provides a broad family of exact analytical solutions, including bright and dark solitons, singular structures, periodic forms, and Jacobi and Weierstrass elliptic solutions, within a single analytical framework. In this way, the study highlights both the reason for selecting the present model and the improvement of the current work relative to earlier investigations.

The contributions of this paper are as follows:

- Utilization of the Improved Modified Extended Tanh-Function (IMETF) method to systematically investigate soliton dynamics in nonlinear Schrödinger equations (NLSE) with self-phase modulation under multiplicative white noise.
- Conducting a study that examines to simultaneously explore the complex interplay between nonlinearity, stochasticity, and self-phase modulation in NLSE without chromatic dispersion, a previously unaddressed scenario.
- Introducing several exact analytical solutions of exact analytical solutions under stochastic influences, including bright, dark, and singular solitons; hyperbolic, periodic, and singular periodic waveforms; and solutions involving Jacobi and Weierstrass elliptic functions.
- contributing to the current literature in current research by advancing theoretical insights into soliton behavior in noisy nonlinear systems and providing robust analytical tools for future exploration.

The self-phase modulated SNLSE without chromatic^1^:

1 $$\begin{aligned} \begin{aligned}&i {\mathfrak {U}}_t+i {\mathfrak {P}}_1 {\mathfrak {U}}_{\text {xxx}}+{\mathfrak {P}}_2 {\mathfrak {U}}_{\text {xt}}+{\mathfrak {P}}_3 {\mathfrak {U}} \left| {\mathfrak {U}}\right| ^{2 n}+i {\mathfrak {P}}_4 {\mathfrak {U}}_x \left| {\mathfrak {U}}\right| ^{2 n} \\&+i {\mathfrak {P}}_5 {\mathfrak {U}} \left| {\mathfrak {U}}\right| ^{2 n}_x-i {\mathfrak {P}}_6 {\mathfrak {U}}_x+\left( \sigma {\mathfrak {U}}-||i \sigma {\mathfrak {P}}_2 {\mathfrak {U}}_x\right) \frac{\text {dW}(t)}{\text {dt}}=0, \end{aligned} \end{aligned}$$

where ${\mathfrak {U}}(x,t)$ is wave profile complex-valued function, the variables *t* and *x* are the temporal and spatial variables, respectively. Furthermore, ${\mathfrak {P}}_1$ and ${\mathfrak {P}}_2$ denote the spatio temporal dispersion mathematical coefficients. In addition, ${\mathfrak {P}}_3$ corresponds to the coefficient of the power-law medium parameter characterizing self-phase modulation. Additionally, the coefficients that account for nonlinear dispersion effects are denoted by, ${\mathfrak {P}}_4$ and ${\mathfrak {P}}_5$. The parameter ${\mathfrak {P}}_6$ is related to inter-modal dispersion.

Additionally, $\sigma$ represents the noise strength coefficient, and $W(t)$ denotes the standard Wiener process. The term “white noise” is mathematically defined as $\frac{dW(t)}{dt}$. The stochastic process is characterized by the following properties:

(i) The function $W(t)$ is continuous for $t \ge 0$.
(ii) The difference $W(t) - W(s)$, for $t > s$, is normally distributed with a mean of zero and a variance of $t - s$.

This process is commonly referred to as Brownian motion^25–27^.

## Basic algorithm of the proposed method

In this section, we provide a brief overview of the *IMETF* approach (see^22–24^). The general form of the nonlinear partial differential equation is given by

2 $$\begin{aligned} \mathbb {F}({\mathfrak {U}}, {\mathfrak {U}}_{t}, {\mathfrak {U}}_{x}, {\mathfrak {U}}_{xx}, \dots ) = 0 \end{aligned}$$

where $\mathbb {F}$ is a polynomial function in ${\mathfrak {U}} = {\mathfrak {U}}(x, t)$ and its different partial derivatives with respect to *t* and *x*, respectively. Also, the highest order derivatives and nonlinear terms are involved.

Let us assume that the wave function solution can be expressed as

3 $$\begin{aligned} {\mathfrak {U}}(x, t) = {\mathfrak {V}}(\zeta ) e^{i({\hat{\chi }})}, \end{aligned}$$

where

4 $$\begin{aligned} \zeta =x-\upsilon t, \end{aligned}$$

Then, Eq.(2) will be converted into a nonlinear ordinary differential equation:

5 $$\begin{aligned} \mathbb {F}({\mathfrak {V}}, {\mathfrak {V}}', {\mathfrak {V}}'',....) = 0. \end{aligned}$$

Assuming the solution of Eq.(5) has in the form:

6 $$\begin{aligned} {\mathfrak {V}}(\zeta ) = \sum _{i=0}^{N} {\mathfrak {A}}_i \Phi ^{i}(\zeta )+\sum _{i=1}^{N} {\mathfrak {B}}_{i} \Phi ^{-i}(\zeta ), \end{aligned}$$

where $$\Phi (\zeta )$$ satisfies

7 $$\begin{aligned} (\Phi ^{\prime })^2 =\sum _{j=0}^{4} \delta _j \Phi ^j. \end{aligned}$$

thus applying the balancing rule in Eq. (5) to evaluate the positive integer *N* .

By comparing the coefficients of the like independent functions to zero with the aid of Eq. (7). This results in a system of algebraic equations that can be solved with the help of Mathematica software packages to evaluate the unknown parameters $\upsilon$ and ${\mathfrak {P}}_j$ for $j=1,2,\dots ,6$, and ${\mathfrak {A}}_i, {\mathfrak {B}}_{i}$ for $i = 0, \pm 1, \pm 2, \dots$. Thus, through the different values of $\delta _0, \delta _1, \delta _2, \delta _3$, and $\delta _4$, we derive the proposed analytical solutions (Table 1).

** Solution Set (I) **: For $\delta _0=\delta _1=\delta _3=0,$ then the solutions are:

8 $$\begin{aligned} \Phi _I(\xi ) = {\left\{ \begin{array}{ll} \sqrt{\frac{-\delta _2}{\delta _4}} \text {sech}\left( \sqrt{-\delta _2\xi }\right) , & \text {if } \delta _2> 0, \delta _4< 0 \\ \sqrt{\frac{\delta _2}{\delta _4}} \sec \left( \sqrt{-\delta _2\xi }\right) , & \text {if } \delta _2 < 0, \delta _4 > 0 \end{array}\right. } \end{aligned}$$

** Solution Set (II) **: For $\delta _1=0,\delta _3=0,\delta _0=\frac{\delta _2^2}{4 \delta _4},$ then the solutions are:

9 $$\begin{aligned} \Phi _{II}(\xi ) = {\left\{ \begin{array}{ll} \sqrt{\frac{\delta _2}{2\delta _4}} \tanh \left( \sqrt{\frac{\delta _2}{2}}\xi \right) , & \text {if } \delta _2 < 0, \delta _4> 0 \\ \sqrt{\frac{\delta _2}{2\delta _4}} \tan \left( \sqrt{\frac{\delta _2}{2}}\xi \right) , & \text {if } \delta _2> 0, \delta _4 > 0 \end{array}\right. } \end{aligned}$$

** Solution Set (III) **:

**Table 1:** The Jacobi elliptic functions for $\delta _1 =\delta _3 = 0$.

$\Phi _{III}(\zeta )$	$\delta _2$	$\delta _4$	$\delta _0$
$\sqrt{\frac{\delta _2\mu ^2}{\delta _4(2-\mu ^2)}} \text {cn}\left( \zeta \sqrt{\frac{\delta _2}{2-\mu ^2}}\right)$	$> 0$	$> 0$	$\frac{\delta _2^2\mu ^2(1-\mu ^2)}{\delta _4(2\mu ^2-1)^2}$
$\sqrt{\frac{-\delta _2}{\delta _4(2-\mu ^2)}} \text {dn}\left( \zeta \sqrt{\frac{\delta _2}{2-\mu ^2}}\right)$	$> 0$	$< 0$	$\frac{\delta _2^2(1-\mu ^2)}{\delta _4(2-\mu ^2)^2}$
$\sqrt{\frac{-\delta _2}{\delta _4(\mu ^2+1)}} \text {sn}\left( \zeta \sqrt{\frac{-\delta _2}{\mu ^2+1}}\right)$	$< 0$	$> 0$	$\frac{\delta _2^2\mu ^2}{\delta _4(\mu ^2+1)^2}$

where $\mu$ is the modulus of Jacobi’s elliptic functions.

**Solution Set (IV) **: For $\delta _0=\delta _1=\delta _4=0,$ then:

10 $$\begin{aligned} \Phi _{IV}(\xi ) = {\left\{ \begin{array}{ll} \frac{\delta _2 \text {sech}^2\left( \frac{\sqrt{\delta _2}}{2}\xi \right) }{\delta _3}, & \text {if } \delta _2 > 0 \\ \frac{\delta _2 \sec ^2\left( \frac{1}{2}\sqrt{-\delta _2}\xi \right) }{\delta _3}, & \text {if } \delta _2 < 0 \\ \frac{1}{\delta _3\xi ^2}, & \text {if } \delta _2 = 0 \end{array}\right. } \end{aligned}$$

** Solution Set (V) **: For $\delta _3=\delta _4=0,$ then:

11 $$\begin{aligned} \Phi _V(\xi ) = {\left\{ \begin{array}{ll} \frac{\delta _1 \sinh \left( 2\sqrt{\delta _2}\xi \right) }{2\delta _2} - \frac{\delta _1}{2\delta _2}, & \text {if } \delta _2> 0, \delta _0 = 0 \\ \frac{\delta _1 \sin \left( \sqrt{-\delta _2}\xi \right) }{2\delta _2} - \frac{\delta _1}{2\delta _2}, & \text {if } \delta _2 < 0, \delta _0 = 0 \\ \exp \left( \sqrt{\delta _2}\xi \right) - \frac{\delta _1}{2\delta _2}, & \text {if } \delta _2 > 0, \delta _0 = \frac{\delta _1^2}{4\delta _2} \end{array}\right. } \end{aligned}$$

** Solution Set (VI) **: For $\delta _1=\delta _3=\delta _4=0,$ then:

12 $$\begin{aligned} \Phi _{VI}(\xi ) = {\left\{ \begin{array}{ll} \sqrt{\frac{\delta _0}{\delta _2}} \sinh \left( \sqrt{\delta _2}\xi \right) , & \text {if } \delta _2> 0, \delta _0> 0 \\ \sqrt{\frac{-\delta _0}{\delta _2}} \sin \left( \sqrt{-\delta _2}\xi \right) , & \text {if } \delta _2 < 0, \delta _0 > 0 \end{array}\right. } \end{aligned}$$

** Solution Set (VII) **: For $\delta _0=\delta _1=0,$ then:

13 $$\begin{aligned} \Phi _{VII}(\xi ) = {\left\{ \begin{array}{ll} \frac{\delta _2 \text {sech}^2\left( \frac{\sqrt{\delta _2}}{2}\xi \right) }{2\sqrt{\delta _2\delta _4} \tanh \left( \frac{\sqrt{\delta _2}}{2}\xi \right) - \delta _3}, & \text {if } \delta _2> 0, \delta _4> 0, \delta _3^2 \ne 4(\delta _2\delta _4) \\ \frac{-\delta _2 \sec ^2\left( \frac{1}{2}\sqrt{-\delta _2}\xi \right) }{2\sqrt{-\delta _2\delta _4} \tan \left( \frac{1}{2}\sqrt{-\delta _2}\xi \right) + \delta _3}, & \text {if } \delta _2 < 0, \delta _4> 0 \\ \frac{1}{2}\sqrt{\frac{\delta _2}{\delta _4}}\left( \tanh \left( \frac{\sqrt{\delta _2}}{2}\xi \right) + 1\right) , & \text {if } \delta _2> 0, \delta _4 > 0, \delta _3^2 = 4(\delta _2\delta _4) \end{array}\right. } \end{aligned}$$

** Solution Set (VIII) **: For $\delta _2=\delta _4=0,$ the Weierstrass Elliptic Doubly Periodic Function solutions are:

$$\begin{aligned} \Phi _{VIII}(\zeta )=\wp \left( \frac{\sqrt{\delta _3} \zeta }{2},-\frac{4 \delta _1}{\delta _3},-\frac{4 \delta _0}{\delta _3}\right) , \hspace{1.5cm}\delta _3>0, \delta _0 \ne 0, \delta _1 \ne 0 \end{aligned}$$

** Solution Set (IX) **: For $\delta _0=\delta _1=\delta _2=0,$ then:

14 $$\begin{aligned} \Phi _{IX}(\xi ) = {\left\{ \begin{array}{ll} \frac{4\delta _3}{\delta _3^2\xi ^2 - 4\delta _4}, & \text {if } \delta _4 \ne 0 \\ \frac{\delta _3}{2\delta _4} \exp \left( \frac{\delta _3\xi }{2\sqrt{-\delta _4}}\right) , & \text {if } \delta _4 < 0 \end{array}\right. } \end{aligned}$$

## Stochastic waveform characterization

In order to find the solutions of Eq.(1), we assume:

15 $$\begin{aligned} {\mathfrak {U}}(x, t) = {\mathfrak {V}}({\hat{\chi }}) e^{i({\hat{\chi }})},~~~~~~{\hat{\chi }} =k x-\upsilon t+\sigma W(t)-\sigma ^{2} t. \end{aligned}$$

The frequency and wave number of the proposed wave function are $k~~and~~\omega$, respectively . Moreover, $\upsilon$ is the wave velocity. Now, by substituting Eq.(15) into Eq.(1) and comparing the coefficients, the real and imaginary parts, we obtain the following:

16 $$\begin{aligned} \begin{aligned}&\left( k^3 {\mathfrak {P}}_1+k \sigma ^2 {\mathfrak {P}}_2+k \omega {\mathfrak {P}}_2+k {\mathfrak {P}}_6+{\widehat{\sigma }} \right) {\mathfrak {V}}(\zeta ) \\&+\left( {\mathfrak {P}}_3-k {\mathfrak {P}}_4\right) {\mathfrak {V}}(\zeta )^{2 n+1}-\left( 3 k {\mathfrak {P}}_1+\upsilon {\mathfrak {P}}_2\right) {\mathfrak {V}}''(\zeta )=0, \end{aligned} \end{aligned}$$

and

17 $$\begin{aligned} \begin{aligned}&\left( 3 k^2 {\mathfrak {P}}_1+k \upsilon {\mathfrak {P}}_2+\upsilon +\sigma ^2 {\mathfrak {P}}_2+\omega {\mathfrak {P}}_2+{\mathfrak {P}}_6\right) {\mathfrak {V}}'(\zeta ) \\&-\left( 2 n {\mathfrak {P}}_5+{\mathfrak {P}}_4\right) {\mathfrak {V}}(\zeta )^{2 n} {\mathfrak {V}}'(\zeta )-{\mathfrak {P}}_1 {\mathfrak {V}}^{(3)}(\zeta )=0. \end{aligned} \end{aligned}$$

Then, the following can be obtained:

18 $$\begin{aligned} \upsilon&= \frac{-3 k^2 {\mathfrak {P}}_1-\sigma ^2 {\mathfrak {P}}_2-\omega {\mathfrak {P}}_2-{\mathfrak {P}}_6}{k {\mathfrak {P}}_2+1}, \nonumber \\ {\mathfrak {P}}_4&= -2 n {\mathfrak {P}}_5. \end{aligned}$$

Now, balancing higher order term ${\mathfrak {V}}''(\zeta )$ and nonlinear term ${\mathfrak {V}}(\zeta )^{2 n+1}$ in Eq. (16) gives the number $N=1/n$.

So, we suppose

19 $$\begin{aligned} {\mathfrak {V}}(\zeta )=\Upsilon (\zeta )^{1/n}. \end{aligned}$$

Based on the conditions (18) and hypothesis (19), the ordinary equation (16) will be transformed to

20 $$\begin{aligned} \begin{aligned}&\Upsilon (\zeta )^2 \Big (k^4 n^2 {\mathfrak {P}}_1 {\mathfrak {P}}_2+k^3 n^2 {\mathfrak {P}}_1+k^2 n^2 \sigma ^2 {\mathfrak {P}}_2^2+k^2 n^2 \omega {\mathfrak {P}}_2^2+k^2 n^2 {\mathfrak {P}}_2 {\mathfrak {P}}_6 \\&+2 k n^2 \sigma ^2 {\mathfrak {P}}_2+2 k n^2 \omega {\mathfrak {P}}_2 +k n^2 {\mathfrak {P}}_6+n^2 \sigma ^2+n^2 \omega \Big ) \\&+\Upsilon (\zeta )^4 \left( 2 k^2 n^3 {\mathfrak {P}}_2 {\mathfrak {P}}_5+2 k n^3 {\mathfrak {P}}_5+k n^2 {\mathfrak {P}}_2 {\mathfrak {P}}_3+n^2 {\mathfrak {P}}_3\right) \\&+\Upsilon '(\zeta )^2 \big (3 k n {\mathfrak {P}}_1-3 k {\mathfrak {P}}_1-n \sigma ^2 {\mathfrak {P}}_2^2-n \omega {\mathfrak {P}}_2^2-n {\mathfrak {P}}_6 {\mathfrak {P}}_2 \\&+\sigma ^2 {\mathfrak {P}}_2^2+\omega {\mathfrak {P}}_2^2+{\mathfrak {P}}_6 {\mathfrak {P}}_2\big ) +\Upsilon (\zeta ) \Upsilon ''(\zeta ) \left( -3 k n {\mathfrak {P}}_1+n \sigma ^2 {\mathfrak {P}}_2^2+n \omega {\mathfrak {P}}_2^2+n {\mathfrak {P}}_6 {\mathfrak {P}}_2\right) =0. \end{aligned} \end{aligned}$$

Balancing the highest-order nonlinear term $\Upsilon (\zeta )^4$ with the highest-order derivative term $\Upsilon (\zeta )\Upsilon ''(\zeta )$ yields the algebraic relation $4N = 2N + 2$, which determines the balancing number $N = 1$,

21 $$\begin{aligned} \Upsilon (\zeta )={\mathfrak {A}}_0+{\mathfrak {A}}_1 \Phi (\zeta )+\frac{{\mathfrak {B}}_1}{\Phi (\zeta )}, \end{aligned}$$

where ${\mathfrak {A}}_0$, ${\mathfrak {A}}_1$, and ${\mathfrak {B}}_1$ are constants.

By substituting Eq. (21) and Eq. (7) into Eq. (20), we obtain a nonlinear system of equations.

Now, by gathering the coefficients of $\Phi (\zeta )$ and equating them to zero, we formulate a solvable system that can be efficiently handled using symbolic computation software such as *Mathematica*. From these solutions, the corresponding forms of Eq. (1) are systematically derived under the specified cases.

**Solution Set I :** If we set $\delta _0=0,\delta _1=0,\delta _3=0$, consequently, the following solutions of Eq.(1) will be:

**(1.1) **:

22 $$\begin{aligned} & {\mathfrak {A}}_0=0, {\mathfrak {A}}_1=\frac{\sqrt{3 \delta _4 k (n+1) {\mathfrak {P}}_1} \sqrt{k^3 {\mathfrak {P}}_1+2 \left( {\widehat{\sigma }} \right) }}{\sqrt{n^2 \left( 2 k n {\mathfrak {P}}_5+{\mathfrak {P}}_3\right) \left( k^3 {\mathfrak {P}}_1+{\widehat{\sigma }} \right) }}, {\mathfrak {B}}_1=0, \delta _2=\frac{n^2 \left( -2 k^3 {\mathfrak {P}}_1+{\widehat{\sigma }} \right) }{3 k {\mathfrak {P}}_1},\nonumber \\ & {\mathfrak {P}}_2=-\frac{3 k^2 {\mathfrak {P}}_1}{k^3 {\mathfrak {P}}_1+{\widehat{\sigma }} }, {\mathfrak {P}}_6=\frac{-3 k^3 {\mathfrak {P}}_1+{\widehat{\sigma }} }{k} \end{aligned}$$

where

23 $$\begin{aligned} {\widehat{\sigma }}=\sigma ^2+\omega . \end{aligned}$$

(1.1.1) Bright soliton solution:

24 $$\begin{aligned} \begin{aligned} {\mathfrak {U}}(x,t) =&\left( \frac{\sqrt{\delta _4 k (n+1) {\mathfrak {P}}_1} \sqrt{k^3 {\mathfrak {P}}_1+2 {\widehat{\sigma }}} \sqrt{-\frac{n^2 (-2 k^3 {\mathfrak {P}}_1+{\widehat{\sigma }})}{\delta _4 k {\mathfrak {P}}_1}}}{\sqrt{n^2 (2 k n {\mathfrak {P}}_5+{\mathfrak {P}}_3) (k^3 {\mathfrak {P}}_1+{\widehat{\sigma }})}} \right. \\&\times \left. \text {sech} \left[ \frac{(x-\upsilon t) \sqrt{\frac{n^2 (-2 k^3 {\mathfrak {P}}_1+{\widehat{\sigma }})}{k {\mathfrak {P}}_1}}}{\sqrt{3}} \right] \right) ^{\frac{1}{n}} e^{i({\hat{\chi }})}, \end{aligned} \end{aligned}$$

when

$$\begin{aligned}\Big (n^2 \left( -2 k^3 {\mathfrak {P}}_1+{\widehat{\sigma }} \right) \Big )\Big (3 k {\mathfrak {P}}_1\Big )>0.\end{aligned}$$

(1.1.2) Singular periodic solution:

25 $$\begin{aligned} \begin{aligned} {\mathfrak {U}}(x,t) =&\left( \frac{\sqrt{\delta _4 k (n+1) {\mathfrak {P}}_1} \sqrt{k^3 {\mathfrak {P}}_1+2 {\widehat{\sigma }}} \sqrt{-\frac{n^2 (-2 k^3 {\mathfrak {P}}_1+{\widehat{\sigma }})}{\delta _4 k {\mathfrak {P}}_1}}}{\sqrt{n^2 (2 k n {\mathfrak {P}}_5+{\mathfrak {P}}_3) (k^3 {\mathfrak {P}}_1+{\widehat{\sigma }})}} \right. \\&\times \left. \sec \left[ \frac{(x-\upsilon t) \sqrt{-\frac{n^2 (-2 k^3 {\mathfrak {P}}_1+{\widehat{\sigma }})}{k {\mathfrak {P}}_1}}}{\sqrt{3}} \right] \right) ^{\frac{1}{n}} e^{i({\hat{\chi }})}, \end{aligned} \end{aligned}$$

when

$$\begin{aligned}\Big (n^2 \left( -2 k^3 {\mathfrak {P}}_1+{\widehat{\sigma }} \right) \Big )\Big (3 k {\mathfrak {P}}_1\Big )<0.\end{aligned}$$

**Solution Set II :** If we set $\delta _1=0,\delta _3=0,\delta _0=\frac{\delta _2^2}{4 \delta _4}$, then, the following solutions of Eq.(1) are obtained :

**(2.1) **:

26 $$\begin{aligned} & {\mathfrak {A}}_0=0, {\mathfrak {B}}_1=0, \delta _2=-\frac{k^2 n^2 \left( -\sqrt{k^2 {\mathfrak {P}}_6^2-6 k {\mathfrak {P}}_6 \left( {\widehat{\sigma }} \right) -3 \left( {\widehat{\sigma }} \right) ^2}+k {\mathfrak {P}}_6+{\widehat{\sigma }} \right) }{6 \left( {\widehat{\sigma }} \right) },\nonumber \\ & {\mathfrak {P}}_1=\frac{-\sqrt{k^2 {\mathfrak {P}}_6^2-6 k {\mathfrak {P}}_6 \left( {\widehat{\sigma }} \right) -3 \left( {\widehat{\sigma }} \right) ^2}-k {\mathfrak {P}}_6+{\widehat{\sigma }} }{2 k^3},\nonumber \\ & {\mathfrak {P}}_2=\frac{\sqrt{k^2 {\mathfrak {P}}_6^2-6 k {\mathfrak {P}}_6 \left( {\widehat{\sigma }} \right) -3 \left( {\widehat{\sigma }} \right) ^2}-k {\mathfrak {P}}_6-3 \sigma ^2-3 \omega }{2 k \left( {\widehat{\sigma }} \right) }, {\mathfrak {P}}_3=-2 k n {\mathfrak {P}}_5. \end{aligned}$$

(2.1.1) Dark soliton solution:

27 $$\begin{aligned} & {\mathfrak {U}}(x,t) = \left( \frac{{\mathfrak {A}}_1 \sqrt{\frac{k^2 n^2 \left( -\sqrt{k^2 {\mathfrak {P}}_6^2-6 k {\mathfrak {P}}_6 \left( {\widehat{\sigma }} \right) -3 \left( {\widehat{\sigma }} \right) ^2}+k {\mathfrak {P}}_6+{\widehat{\sigma }} \right) }{\delta _4 \left( {\widehat{\sigma }} \right) }}}{2 \sqrt{3}} \right. \nonumber \\ & \quad \times \tanh \left. \left[ \frac{(x-\upsilon t) \sqrt{\frac{k^2 n^2 \left( -\sqrt{k^2 {\mathfrak {P}}_6^2-6 k {\mathfrak {P}}_6 \left( {\widehat{\sigma }} \right) -3 \left( {\widehat{\sigma }} \right) ^2}+k {\mathfrak {P}}_6+{\widehat{\sigma }} \right) }{{\widehat{\sigma }} }}}{2 \sqrt{3}}\right] \right) ^\frac{1}{n} e^{i({\hat{\chi }})}, \end{aligned}$$

when

$$\begin{aligned}\Big (k^2 n^2 \left( -\sqrt{k^2 {\mathfrak {P}}_6^2-6 k {\mathfrak {P}}_6 \left( {\widehat{\sigma }} \right) -3 \left( {\widehat{\sigma }} \right) ^2}+k {\mathfrak {P}}_6+{\widehat{\sigma }} \right) \Big )\Big (6 \left( {\widehat{\sigma }} \right) \Big )>0.\end{aligned}$$

(2.1.2) Singular periodic solution:

28 $$\begin{aligned} & {\mathfrak {U}}(x,t) = \left( \frac{{\mathfrak {A}}_1 \sqrt{-\frac{k^2 n^2 \left( -\sqrt{k^2 {\mathfrak {P}}_6^2-6 k {\mathfrak {P}}_6 \left( {\widehat{\sigma }} \right) -3 \left( {\widehat{\sigma }} \right) ^2}+k {\mathfrak {P}}_6+{\widehat{\sigma }} \right) }{\delta _4 \left( {\widehat{\sigma }} \right) }}}{2 \sqrt{3}} \right. \nonumber \\ & \quad \times \left. \tan \left[ \frac{(x-\upsilon t) \sqrt{-\frac{k^2 n^2 \left( -\sqrt{k^2 {\mathfrak {P}}_6^2-6 k {\mathfrak {P}}_6 \left( {\widehat{\sigma }} \right) -3 \left( {\widehat{\sigma }} \right) ^2}+k {\mathfrak {P}}_6+{\widehat{\sigma }} \right) }{{\widehat{\sigma }} }}}{2 \sqrt{3}}\right] \right) ^\frac{1}{n} e^{i({\hat{\chi }})}, \end{aligned}$$

when

$$\begin{aligned}\Big (k^2 n^2 \left( -\sqrt{k^2 {\mathfrak {P}}_6^2-6 k {\mathfrak {P}}_6 \left( {\widehat{\sigma }} \right) -3 \left( {\widehat{\sigma }} \right) ^2}+k {\mathfrak {P}}_6+{\widehat{\sigma }} \right) \Big )\Big (6 \left( {\widehat{\sigma }} \right) \Big )<0.\end{aligned}$$

**Solution Set III :** If we set $\delta _1=0,\delta _3=0$, then Jacobi elliptic functions solutions of Eq.(1) are obtained :

**(3.1) **:

29 $$\begin{aligned} {\mathfrak {A}}_0&= 0, \quad {\mathfrak {B}}_1 = \frac{\sqrt{\delta _0} {\mathfrak {A}}_1}{\sqrt{\delta _4}}, \quad {\mathfrak {P}}_2 = -\frac{3 k^2 {\mathfrak {P}}_1}{k^3 {\mathfrak {P}}_1+{\widehat{\sigma }} }, \quad {\mathfrak {P}}_3 = -2 k n {\mathfrak {P}}_5, \nonumber \\ {\mathfrak {P}}_6&= -\frac{k^6 {\mathfrak {P}}_1^2-k^3 {\mathfrak {P}}_1 {\widehat{\sigma }} + {\widehat{\sigma }}^2}{k (k^3 {\mathfrak {P}}_1+{\widehat{\sigma }})}. \end{aligned}$$

(3.1.1) Setting $$\delta _2>0, \delta _4<0, \delta _0=\frac{\delta _2^2 \mu ^2 (1-\mu ^2)}{\delta _4 (2 \mu ^2-1)^2}$$:

30 $$\begin{aligned} \begin{aligned} {\mathfrak {U}}(x,t) =&\left( {\mathfrak {A}}_1 \sqrt{\frac{\delta _2 \mu ^2}{\delta _4 (1-2 \mu ^2)}} \text {cn}\left[ (x-\upsilon t) \sqrt{-\frac{\delta _2}{1-2 \mu ^2}}\right] \right. \\&\left. + {\mathfrak {A}}_1 \sqrt{1-2 \mu ^2} \sqrt{\frac{\delta _2 (1-\mu ^2)}{\delta _4 (2 \mu ^2-1)^2}} \text {nc}\left[ (x-\upsilon t) \sqrt{-\frac{\delta _2}{1-2 \mu ^2}}\right] \right) ^{\frac{1}{n}} e^{i({\hat{\chi }})}. \end{aligned} \end{aligned}$$

When $$\mu =1$$:

31 $$\begin{aligned} {\mathfrak {U}}(x,t) = \left( \sqrt{-\frac{\delta _2}{\delta _4}} {\mathfrak {A}}_1 \text {sech}\left[ \sqrt{\delta _2} (x-\upsilon t) \right] \right) ^{\frac{1}{n}} e^{i({\hat{\chi }})}. \end{aligned}$$

(3.1.2) Setting $$\delta _2>0, \delta _4<0, \delta _0=\frac{\delta _2^2 (1-\mu ^2)}{\delta _4 (2-\mu ^2)^2}$$:

32 $$\begin{aligned} \begin{aligned} {\mathfrak {U}}(x,t) =&\left( {\mathfrak {A}}_1 \sqrt{-\frac{\mu ^2}{\delta _4 (2-\mu ^2)}} \text {dn}\left[ (x-\upsilon t) \sqrt{\frac{\delta _2}{2-\mu ^2}}\right] \right. \\&\left. + \frac{{\mathfrak {A}}_1 \sqrt{\frac{\delta _2^2 (1-\mu ^2)}{\delta _4 (2-\mu ^2)^2}}}{\sqrt{\delta _4} \sqrt{-\frac{\mu ^2}{\delta _4 (2-\mu ^2)}}} \text {nd}\left[ (x-\upsilon t) \sqrt{\frac{\delta _2}{2-\mu ^2}}\right] \right) ^{\frac{1}{n}} e^{i({\hat{\chi }})}. \end{aligned} \end{aligned}$$

When $$\mu =1$$:

33 $$\begin{aligned} {\mathfrak {U}}(x,t) = \left( \sqrt{-\frac{1}{\delta _4}} {\mathfrak {A}}_1 \text {sech}\left[ \sqrt{\delta _2} (x-\upsilon t) \right] \right) ^{\frac{1}{n}} e^{i({\hat{\chi }})}. \end{aligned}$$

(3.1.3) Setting $$\delta _2<0, \delta _4>0, \delta _0=\frac{\delta _2^2 \mu ^2}{\delta _4 (\mu ^2+1)^2}$$:

34 $$\begin{aligned} \begin{aligned} {\mathfrak {U}}(x,t) =&\left( {\mathfrak {A}}_1 \sqrt{-\frac{\delta _2 \mu ^2}{\delta _4 (\mu ^2+1)}} \text {sn}\left( (x-\upsilon t) \sqrt{-\frac{\delta _2}{\mu ^2+1}}\right) \right. \\&\left. + \frac{{\mathfrak {A}}_1 \sqrt{\frac{\delta _2^2 \mu ^2}{\delta _4 (\mu ^2+1)^2}}}{\sqrt{\delta _4} \sqrt{-\frac{\delta _2 \mu ^2}{\delta _4 (\mu ^2+1)}}} \text {ns}\left[ (x-\upsilon t) \sqrt{-\frac{\delta _2}{\mu ^2+1}}\right] \right) ^{\frac{1}{n}} e^{i({\hat{\chi }})}. \end{aligned} \end{aligned}$$

when $$\mu =1$$,

35 $$\begin{aligned} & {\mathfrak {U}}(x,t) = \left( \frac{\sqrt{-\frac{\delta _2}{\delta _4}} {\mathfrak {A}}_1 \tanh \left[ \frac{\sqrt{-\delta _2} (x-\upsilon t) }{\sqrt{2}}\right] }{\sqrt{2}}+\frac{\sqrt{\frac{\delta _2^2}{\delta _4}} {\mathfrak {A}}_1 \coth \left[ \frac{\sqrt{-\delta _2} (x-\upsilon t) }{\sqrt{2}}\right] }{\sqrt{2} \sqrt{-\frac{\delta _2}{\delta _4}} \sqrt{\delta _4}}\right) ^\frac{1}{n} e^{i({\hat{\chi }})}. \end{aligned}$$

**Solution Set IV :** If we set $\delta _0=0,\delta _1=0,\delta _4=0$, then the following solutions of Eq.(1) are obtained:

**(4.1) **:

36 $$\begin{aligned} & {\mathfrak {A}}_0=0, {\mathfrak {A}}_1=0, \delta _2=\frac{n^2 \left( -2 k^3 {\mathfrak {P}}_1+{\widehat{\sigma }} \right) }{3 k {\mathfrak {P}}_1}, {\mathfrak {P}}_2=-\frac{3 k^2 {\mathfrak {P}}_1}{k^3 {\mathfrak {P}}_1+{\widehat{\sigma }} }, {\mathfrak {P}}_3=-2 k n {\mathfrak {P}}_5,\nonumber \\ & {\mathfrak {P}}_6=\frac{k^6 \left( -{\mathfrak {P}}_1^2\right) +k^3 \sigma ^2 {\mathfrak {P}}_1+k^3 \omega {\mathfrak {P}}_1-\sigma ^4-2 \sigma ^2 \omega -\omega ^2}{k \left( k^3 {\mathfrak {P}}_1+{\widehat{\sigma }} \right) }. \end{aligned}$$

(4.1.1) Hyperbolic soliton solution:

37 $$\begin{aligned} & {\mathfrak {U}}(x,t) = \left( -\frac{3 \delta _3 k {\mathfrak {B}}_1 {\mathfrak {P}}_1 \cosh ^2\left[ \frac{(x-\upsilon t) \sqrt{\frac{n^2 \left( -2 k^3 {\mathfrak {P}}_1+{\widehat{\sigma }} \right) }{k {\mathfrak {P}}_1}}}{2 \sqrt{3}}\right] }{n^2 \left( -2 k^3 {\mathfrak {P}}_1+{\widehat{\sigma }} \right) }\right) ^\frac{1}{n} e^{i({\hat{\chi }})}, \end{aligned}$$

when

$$\begin{aligned}\Big (n^2 \left( -2 k^3 {\mathfrak {P}}_1+{\widehat{\sigma }} \right) \Big )\Big (3 k {\mathfrak {P}}_1\Big )>0.\end{aligned}$$

(4.1.2) Trigonometric solution:

38 $$\begin{aligned} & {\mathfrak {U}}(x,t) = \left( -\frac{3 \delta _3 k {\mathfrak {B}}_1 {\mathfrak {P}}_1 \cos ^2\left[ \frac{(x-\upsilon t) \sqrt{-\frac{n^2 \left( -2 k^3 {\mathfrak {P}}_1+{\widehat{\sigma }} \right) }{k {\mathfrak {P}}_1}}}{2 \sqrt{3}}\right] }{n^2 \left( -2 k^3 {\mathfrak {P}}_1+{\widehat{\sigma }} \right) }\right) ^\frac{1}{n} e^{i({\hat{\chi }})}, \end{aligned}$$

when

$$\begin{aligned}\Big (n^2 \left( -2 k^3 {\mathfrak {P}}_1+{\widehat{\sigma }} \right) \Big )\Big (3 k {\mathfrak {P}}_1\Big )<0.\end{aligned}$$

**Solution Set V :** If we set $\delta _1=0,\delta _3=0,\delta _4=0$, thus the following solutions of Eq.(1) are acquired.

**(5.1) **:

39 $$\begin{aligned} {\mathfrak {A}}_0&= 0, \quad {\mathfrak {A}}_1=0, \quad {\mathfrak {B}}_1 = \frac{\sqrt{\delta _0} \sqrt{n+1} \sqrt{3 k^2 {\mathfrak {P}}_1-{\mathfrak {P}}_2 {\widehat{\sigma }}}}{\sqrt{k n^2 (2 k n {\mathfrak {P}}_5+{\mathfrak {P}}_3)}}, \nonumber \\ \delta _2&= \frac{k n^2 ((k {\mathfrak {P}}_2+2) {\widehat{\sigma }} - 2 k^3 {\mathfrak {P}}_1)}{3 k^2 {\mathfrak {P}}_1-{\mathfrak {P}}_2 {\widehat{\sigma }}}, \quad {\mathfrak {P}}_6 = \frac{-3 k^3 {\mathfrak {P}}_1+{\widehat{\sigma }} }{k}. \end{aligned}$$

(5.1.1) Singular soliton solution:

40 $$\begin{aligned} {\mathfrak{U}}(x,t) = & \left( {\frac{{\sqrt {n + 1} \sqrt {3k^{2} {\mathfrak{P}}_{1} - {\mathfrak{P}}_{2} \left( {\hat{\sigma }} \right)} {\mathrm{csch}}\left[ {(x - \upsilon t)\sqrt {\frac{{kn^{2} \left( {\left( {k{\mathfrak{P}}_{2} + 2} \right)\left( {\hat{\sigma }} \right) - 2k^{3} {\mathfrak{P}}_{1} } \right)}}{{3k^{2} {\mathfrak{P}}_{1} - {\mathfrak{P}}_{2} \left( {\hat{\sigma }} \right)}}} } \right]}}{{\sqrt {kn^{2} \left( {2kn{\mathfrak{P}}_{5} + {\mathfrak{P}}_{3} } \right)} \sqrt {\frac{{3k^{2} {\mathfrak{P}}_{1} - {\mathfrak{P}}_{2} \left( {\hat{\sigma }} \right)}}{{kn^{2} \left( {\left( {k{\mathfrak{P}}_{2} + 2} \right)\left( {\hat{\sigma }} \right) - 2k^{3} {\mathfrak{P}}_{1} } \right)}}} }}} \right)^{{\frac{1}{n}}} \\ & \times \;e^{{i(\hat{\chi })}} , \\ \end{aligned}$$

when

$$\begin{aligned}\Big (k n^2 \left( \left( k {\mathfrak {P}}_2+2\right) \left( {\widehat{\sigma }} \right) -2 k^3 {\mathfrak {P}}_1\right) \Big )\Big (3 k^2 {\mathfrak {P}}_1-{\mathfrak {P}}_2 \left( {\widehat{\sigma }} \right) \Big )>0.\end{aligned}$$

(5.1.2) Singular periodic solution:

41 $$\begin{aligned} {\mathfrak {U}}(x,t) = & \left( \frac{\sqrt{n+1} \sqrt{3 k^2 {\mathfrak {P}}_1-{\mathfrak {P}}_2 \left( {\widehat{\sigma }} \right) } \csc \left[ (x-\upsilon t) \sqrt{-\frac{k n^2 \left( \left( k {\mathfrak {P}}_2+2\right) \left( {\widehat{\sigma }} \right) -2 k^3 {\mathfrak {P}}_1\right) }{3 k^2 {\mathfrak {P}}_1-{\mathfrak {P}}_2 \left( {\widehat{\sigma }} \right) }}\right] }{\sqrt{k n^2 \left( 2 k n {\mathfrak {P}}_5+{\mathfrak {P}}_3\right) } \sqrt{-\frac{3 k^2 {\mathfrak {P}}_1-{\mathfrak {P}}_2 \left( {\widehat{\sigma }} \right) }{k n^2 \left( \left( k {\mathfrak {P}}_2+2\right) \left( {\widehat{\sigma }} \right) -2 k^3 {\mathfrak {P}}_1\right) }}}\right) ^\frac{1}{n} \nonumber \\ & \times\;e^{i({\hat{\chi }})}, \end{aligned}$$

when

$$\begin{aligned}\Big (k n^2 \left( \left( k {\mathfrak {P}}_2+2\right) \left( {\widehat{\sigma }} \right) -2 k^3 {\mathfrak {P}}_1\right) \Big )\Big (3 k^2 {\mathfrak {P}}_1-{\mathfrak {P}}_2 \left( {\widehat{\sigma }} \right) \Big )<0.\end{aligned}$$

**Solution Set VI :** If we set $\delta _0=0,\delta _1=0$, then the following solutions of Eq.(1) are obtained:

**(6.1) **:

42 $$\begin{aligned} & {\mathfrak {A}}_1=0, {\mathfrak {B}}_1=\frac{2 n^2 {\mathfrak {A}}_0 \left( k {\mathfrak {P}}_2+1\right) \left( k^3 {\mathfrak {P}}_1+k {\mathfrak {P}}_2 \left( {\widehat{\sigma }} \right) +k {\mathfrak {P}}_6+{\widehat{\sigma }} \right) }{\delta _3 \left( 3 k {\mathfrak {P}}_1-{\mathfrak {P}}_2 \left( {\mathfrak {P}}_2 \left( {\widehat{\sigma }} \right) +{\mathfrak {P}}_6\right) \right) },\nonumber \\ & \delta _2=\frac{n^2 \left( k {\mathfrak {P}}_2+1\right) \left( k^3 {\mathfrak {P}}_1+k {\mathfrak {P}}_2 \left( {\widehat{\sigma }} \right) +k {\mathfrak {P}}_6+{\widehat{\sigma }} \right) }{3 k {\mathfrak {P}}_1-{\mathfrak {P}}_2 \left( {\mathfrak {P}}_2 \left( {\widehat{\sigma }} \right) +{\mathfrak {P}}_6\right) },\nonumber \\ & \delta _4=\frac{\delta _3^2 \left( 3 k {\mathfrak {P}}_1-{\mathfrak {P}}_2 \left( {\mathfrak {P}}_2 \left( {\widehat{\sigma }} \right) +{\mathfrak {P}}_6\right) \right) }{4 n^2 \left( k {\mathfrak {P}}_2+1\right) \left( k^3 {\mathfrak {P}}_1+k {\mathfrak {P}}_2 \left( {\widehat{\sigma }} \right) +k {\mathfrak {P}}_6+{\widehat{\sigma }} \right) }, {\mathfrak {P}}_3=-2 k n {\mathfrak {P}}_5. \end{aligned}$$

(6.1.1) Singular soliton solution:

43 $$\begin{aligned} \begin{aligned} {\mathfrak {U}}(x,t) = &\Bigg ({\mathfrak {A}}_0+2 {\mathfrak {A}}_0 \cosh ^2\left[ \frac{1}{2} (x-\upsilon t) \sqrt{\frac{n^2 (k {\mathfrak {P}}_2+1) (k^3 {\mathfrak {P}}_1+k {\mathfrak {P}}_2 {\widehat{\sigma }} +k {\mathfrak {P}}_6+{\widehat{\sigma }})}{3 k {\mathfrak {P}}_1-{\mathfrak {P}}_2 ({\mathfrak {P}}_2 {\widehat{\sigma }} +{\mathfrak {P}}_6)}}\right] \\ & \times \left( \tanh \left[ \frac{1}{2} (x-\upsilon t) \sqrt{\frac{n^2 (k {\mathfrak {P}}_2+1) (k^3 {\mathfrak {P}}_1+k {\mathfrak {P}}_2 {\widehat{\sigma }} +k {\mathfrak {P}}_6+{\widehat{\sigma }})}{3 k {\mathfrak {P}}_1-{\mathfrak {P}}_2 ({\mathfrak {P}}_2 {\widehat{\sigma }} +{\mathfrak {P}}_6)}}\right] -1\right) \Bigg )^{\frac{1}{n}} e^{i({\hat{\chi }})}, \end{aligned} \end{aligned}$$

when

$$\begin{aligned}\Big (n^2 \left( k {\mathfrak {P}}_2+1\right) \left( k^3 {\mathfrak {P}}_1+k {\mathfrak {P}}_2 \left( {\widehat{\sigma }} \right) +k {\mathfrak {P}}_6+{\widehat{\sigma }} \right) \Big )\Big (3 k {\mathfrak {P}}_1-{\mathfrak {P}}_2 \left( {\mathfrak {P}}_2 \left( {\widehat{\sigma }} \right) +{\mathfrak {P}}_6\right) \Big )>0.\end{aligned}$$

(6.1.2) Singular periodic solution:

44 $$\begin{aligned} {\mathfrak {U}}(x,t) = & \Big ({\mathfrak {A}}_0-\frac{2 {\mathfrak {A}}_0 \cos ^2\left[ \frac{1}{2} (x-\upsilon t) \sqrt{-\frac{n^2 \left( k {\mathfrak {P}}_2+1\right) \left( k^3 {\mathfrak {P}}_1+k {\mathfrak {P}}_2 \left( {\widehat{\sigma }} \right) +k {\mathfrak {P}}_6+{\widehat{\sigma }} \right) }{3 k {\mathfrak {P}}_1-{\mathfrak {P}}_2 \left( {\mathfrak {P}}_2 \left( {\widehat{\sigma }} \right) +{\mathfrak {P}}_6\right) }}\right] }{\delta _3} \nonumber \\ & \times \left( \sqrt{-\delta _3^2} \tan \left[ \frac{1}{2} (x-\upsilon t) \sqrt{-\frac{n^2 \left( k {\mathfrak {P}}_2+1\right) \left( k^3 {\mathfrak {P}}_1+k {\mathfrak {P}}_2 \left( {\widehat{\sigma }} \right) +k {\mathfrak {P}}_6+{\widehat{\sigma }} \right) }{3 k {\mathfrak {P}}_1-{\mathfrak {P}}_2 \left( {\mathfrak {P}}_2 \left( {\widehat{\sigma }} \right) +{\mathfrak {P}}_6\right) }}\right] +\delta _3\right) \Big )^\frac{1}{n} \nonumber \\ & \times e^{i({\hat{\chi }})}, \end{aligned}$$

when

$$\begin{aligned}\Big (n^2 \left( k {\mathfrak {P}}_2+1\right) \left( k^3 {\mathfrak {P}}_1+k {\mathfrak {P}}_2 \left( {\widehat{\sigma }} \right) +k {\mathfrak {P}}_6+{\widehat{\sigma }} \right) \Big )\Big (3 k {\mathfrak {P}}_1-{\mathfrak {P}}_2 \left( {\mathfrak {P}}_2 \left( {\widehat{\sigma }} \right) +{\mathfrak {P}}_6\right) \Big )<0.\end{aligned}$$

**Solution Set VII :** If we set $\delta _2=0,\delta _4=0$, then the following solution of Eq.(1) is obtained:

**(7.1) **:

45 $$\begin{aligned} & {\mathfrak {A}}_1=-\frac{4 k^2 n^2 {\mathfrak {A}}_0 \left( k {\mathfrak {P}}_2+1\right) }{\delta _1 (n-2)};{\mathfrak {B}}_1=0, {\mathfrak {P}}_1=\frac{-\sigma ^2 {\mathfrak {P}}_2-\omega {\mathfrak {P}}_2}{k^2 \left( k {\mathfrak {P}}_2+3\right) }, {\mathfrak {P}}_3=-2 k n {\mathfrak {P}}_5,\nonumber \\ & {\mathfrak {P}}_6=-\frac{\left( k^2 {\mathfrak {P}}_2^2+3 k {\mathfrak {P}}_2+3\right) \left( {\widehat{\sigma }} \right) }{k \left( k {\mathfrak {P}}_2+3\right) }. \end{aligned}$$

(7.1.1) Solution in Terms of Weierstrass Elliptic Functions :

46 $$\begin{aligned} & {\mathfrak {U}}(x,t) = \left( {\mathfrak {A}}_0-\frac{4 k^2 n^2 {\mathfrak {A}}_0 \left( k {\mathfrak {P}}_2+1\right) \wp \left( \frac{\sqrt{\delta _3} \zeta }{2},-\frac{4 \delta _1}{\delta _3},-\frac{4 \delta _0}{\delta _3}\right) }{\delta _1 (n-2)}\right) ^\frac{1}{n} e^{i({\hat{\chi }})}, \end{aligned}$$

## Visual graphs and analysis for some solutions

Two soliton solutions that were produced from the PRNLSE using the IMETF technique are shown graphically in this section. The bright soliton solution ${\mathfrak {U}}_{1.1.1}$ (Eq. 16) and the dark soliton solution ${\mathfrak {U}}_{2.1.1}$ (Eq. 18) have been represented. The graphical analysis systematically examines the influence of multiplicative white noise on both solution families across four distinct noise intensity regimes: $\sigma = 0$ (deterministic case), $\sigma = 0.2$ (weak noise), $\sigma = 0.6$ (moderate noise), and $\sigma = 1.0$ (strong noise). For each solution type, we present both two-dimensional cross-sectional profiles and three-dimensional surface plots of the real and imaginary parts, providing complete visualization of the complex-valued wave functions and their stochastic evolution. The comparative analysis reveals fundamental differences in how bright and dark solitons respond to multiplicative white noise perturbations, highlighting the amplitude-dependent nature of stochastic effects and their distinct impact on localized versus depleted wave structures.

**Fig. 1:**
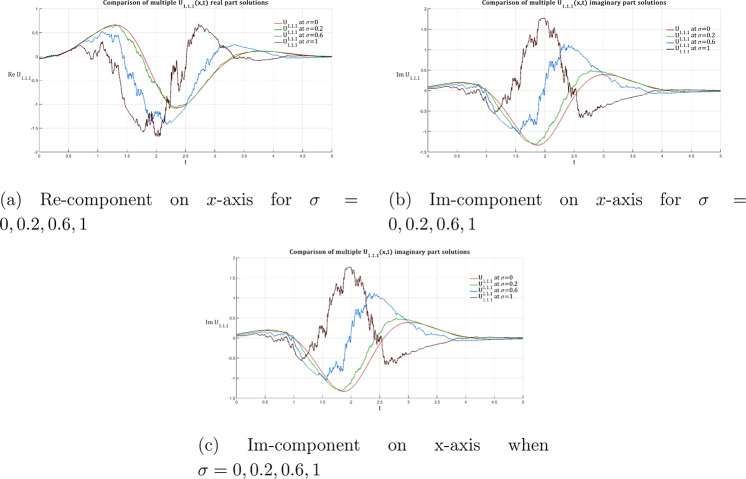
Evolution of the 2D bright soliton profile ${\mathfrak {U}}_{1.1.1}$ with increasing noise intensity according to Eq. (24), illustrating the transition from deterministic ($\sigma =0$) to highly stochastic ($\sigma =1$) regimes. System parameters are fixed as ${\mathfrak {P}}_1 = 0.2$, ${\mathfrak {P}}_3 = -0.8$, ${\mathfrak {P}}_5 = -1.2$, $\omega = 2$, $k = 1$, $n = 1$, $\upsilon = 1$, and $\delta _4 = -2$. This figure highlights the impact of noise on soliton profiles within these specified conditions.

**Fig. 2:**
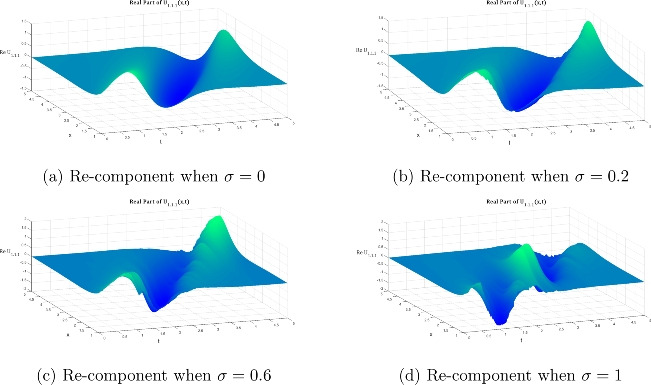
Volumetric visualization of the real part of the bright soliton ${\mathfrak {U}}_{1.1.1}$: noise-induced morphological transitions across $\sigma \in \{0, 0.2, 0.6, 1\}$ in Equation (24) under fixed parametric constraints (${\mathfrak {P}}_1 = 0.2$, ${\mathfrak {P}}_3 = -0.8$, ${\mathfrak {P}}_5 = -1.2$, $\omega = 2$, $k = 1$, $n = 1$, $\upsilon = 1$, $\delta _4 = -2$).

**Fig. 3:**
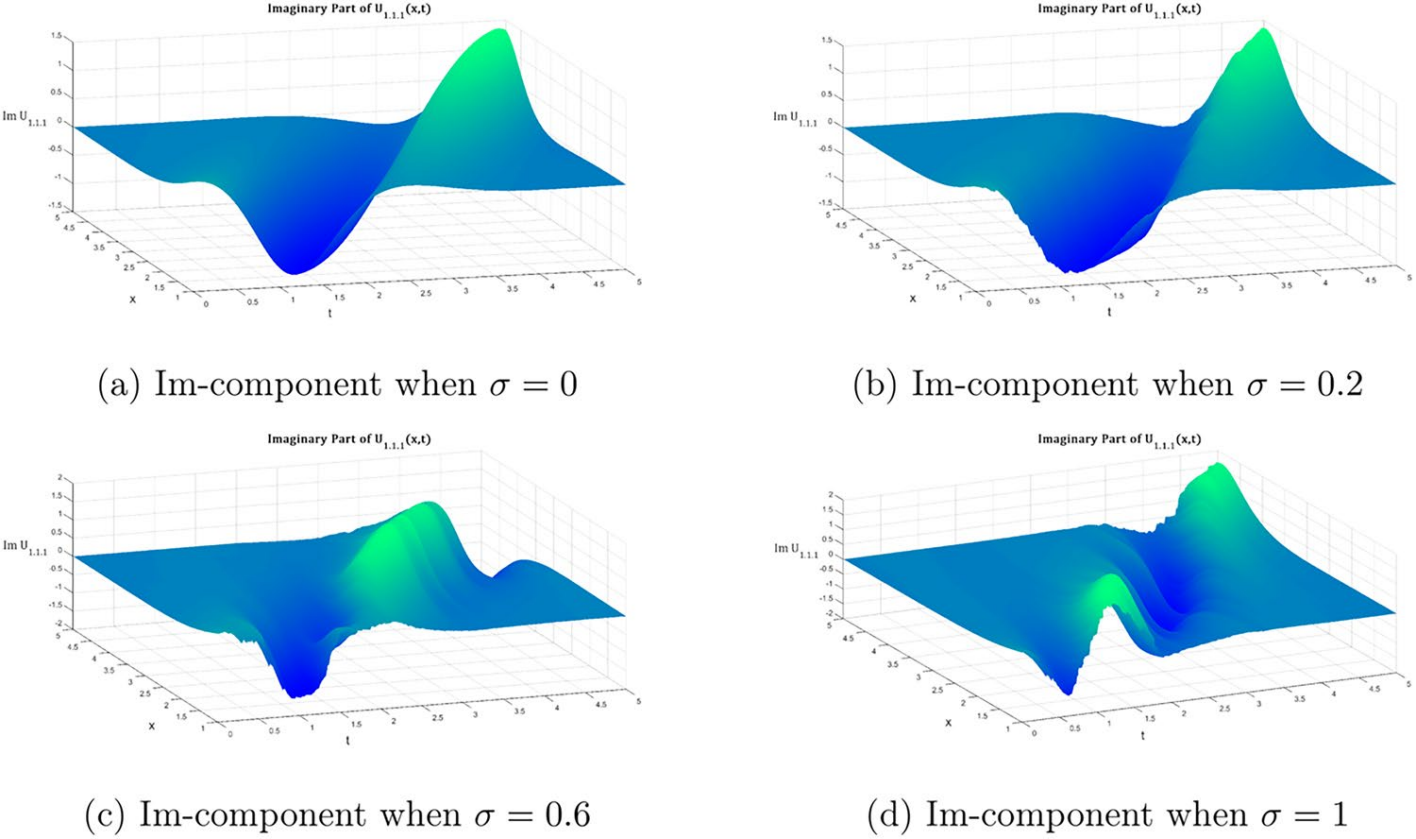
The same as figure 2 fut for the imaginary part.

Figures 1, 2, and 3 illustrate the two- and three-dimensional behavior of the bright soliton solution $\hbox {U}_{1.1.1}$ under varying noise intensities, revealing distinct noise-induced modifications in both real and imaginary components. In the deterministic case ($\sigma = 0$), the real component exhibits a characteristic bell-shaped profile with smooth, symmetric decay and maximum amplitude around 0.8. Under weak noise conditions ($\sigma = 0.2$), minor amplitude fluctuations are introduced while preserving the overall soliton structure. As the noise intensity increases to moderate levels ($\sigma = 0.6$), the solution displays significant amplitude modulation with increased irregularities and slight broadening. This progression culminates in dramatic structural changes at strong noise levels ($\sigma = 1.0$), characterized by multiple local extrema and substantial amplitude variations that fundamentally alter the soliton’s coherent structure.

The imaginary component behavior demonstrates the antisymmetric structure typical of bright solitons, with balanced positive and negative lobes in the deterministic case. Progressive noise effects show increasing phase distortion and loss of symmetry, with the strong noise case ($\sigma = 1.0$) exhibiting severe structural deformation that fundamentally alters the phase characteristics of the soliton. The three-dimensional surface plots provide comprehensive spatial visualization of this noise-induced deformation. The deterministic real component surface ($\sigma = 0$) shows a smooth, localized peak characteristic of bright soliton focusing. However, progressive noise introduction creates increasing surface roughness and amplitude fluctuations that culminate in highly irregular surfaces with multiple peaks and valleys at high noise levels ($\sigma = 1.0$).

Similarly, the imaginary component evolution reveals how the clean antisymmetric structure in the deterministic case progressively degrades with increasing noise. While moderate noise levels introduce surface undulations that maintain recognizable soliton features, strong noise completely disrupts the phase coherence, resulting in chaotic surface topology that destroys the fundamental soliton characteristics.

**Fig. 4:**
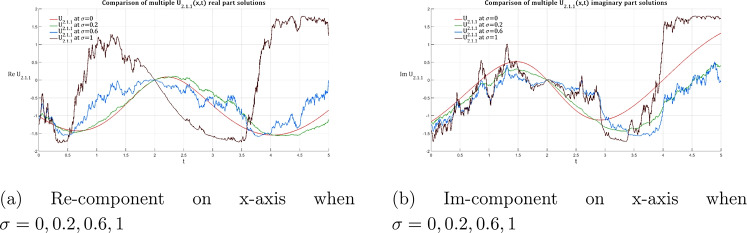
Evolution of the $2\textrm{D}$ dark soliton profile ${\mathfrak {U}}_{2.1.1}$ under increasing noise intensity: Equation (27) with $\sigma$ progressing from deterministic (0) to highly stochastic (1) regimes, maintaining fixed system parameters (${\mathfrak {P}}_5 = 0.2$, ${\mathfrak {P}}_6 = 7$, $\omega = 1$, $k = 2$, $n = 1$, ${\mathfrak {A}}_1=1.8$, $\upsilon = 1$, $\delta _4 = 2$).

**Fig. 5:**
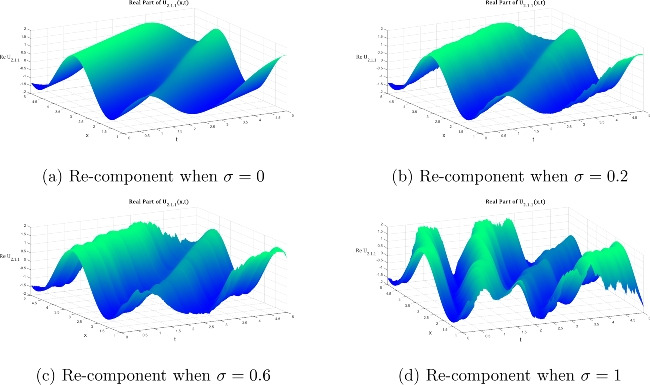
Volumetric visualization of the real part of the dark soliton ${\mathfrak {U}}_{2.1.1}$ : noise-induced morphological transitions across $\sigma \in \{0, 0.2, 0.6, 1\}$ in Equation (27) under fixed parametric constraints (${\mathfrak {P}}_5 = 0.2$, ${\mathfrak {P}}_6 = 7$, $\omega = 1$, $k = 2$, $n = 1$, ${\mathfrak {A}}_1=1.8$, $\upsilon = 1$, $\delta _4 = 2$)..

**Fig. 6:**
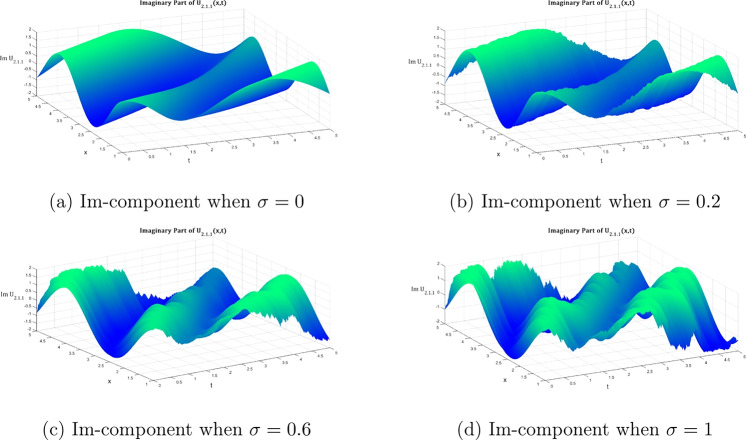
The same as Fig. 5 but for the imaginary part.

Figures 4, 5, and 6 demonstrate that dark solitons ${\mathfrak {U}}_{2.1.1}$ exhibit fundamentally different noise response characteristics compared to their bright counterparts. The deterministic case ($\sigma = 0$) shows the characteristic dark soliton profile with central depletion flanked by elevated background levels. Noise effects demonstrate progressive filling of the central depression and background level fluctuations that intensify with increasing noise intensity. At strong noise levels ($\sigma = 1.0$), the solution undergoes complete loss of the dark soliton structure, replaced by multiple oscillatory features that bear no resemblance to the original coherent structure.

The imaginary component of dark solitons exhibits smooth phase variation across the soliton profile in the deterministic case but shows progressive degradation with increasing phase fluctuations that ultimately destroy the coherent dark soliton structure. This phase degradation is particularly pronounced in dark solitons due to their inherent dependence on phase coherence for maintaining the characteristic depletion structure. The three-dimensional representation of dark solitons reveals unique noise-induced phenomena that distinguish them from bright solitons. The real component surface evolution shows how the characteristic dark soliton valley surrounded by elevated regions in the deterministic case becomes progressively filled as noise increases, with surface irregularities that grow in magnitude until high noise levels completely eliminate the localized depletion, replacing it with chaotic surface variations.

The imaginary component surface evolution demonstrates how the clean phase structure in the deterministic case becomes increasingly distorted. Moderate noise creates surface ripples that grow into major structural disruptions, while strong noise results in complete loss of the organized phase pattern. This behavior reflects the fundamental vulnerability of dark solitons to background noise, which disrupts the delicate balance required to maintain the coherent depletion structure.

A comprehensive comparison between the present investigation and the results obtained in Ref.^26^ is outlined in Table 2 While both studies analyze PRNLSE under multiplicative white noise, they explore distinct physical regimes and employ different analytical strategies. The current work focuses on the dispersionless limit governed by self-phase modulation and utilizes IMETF method to derive robust elliptic and soliton solutions. Conversely, Ref.^26^ investigates spatial noise intensity using a hybrid of three integration techniques, Logarithmic transformation, $(G'/G, 1/G)$-expansion, and generalized Kudryashov methods to extract chaotic patterns and specific waveforms like M-shaped solitons. This distinction highlights the unique contribution of our study in establishing noise resilience thresholds for optical solitons.

**Table 2:** Comparison of Analytical Studies on SNLSE.

Comparison point	The current work	Ref.^25^
Mathematical model & noise	Investigates the SNLSE with Self-Phase Modulation (SPM) in the dispersionless regime. The noise is defined as multiplicative white noise (*dW*(*t*)/*dt*).	Investigates the SNLSE incorporating spatial noise intensity. The noise model involves multiplicative white noise driven by Brownian motion ($E_t$) and a spatial intensity parameter ($\sigma _1$).
Analytical methodologies	Utilizes a single unified approach: the Improved Modified Extended Tanh-Function (IMETF) method.	Employs a combination of three techniques: the Logarithmic transformation, the $(G'/G, 1/G)$-expansion method, and the Generalized Kudryashov method (gKM).
Derived exact solutions	Derives bright and dark solitons, singular solitons, singular periodic structures, and solutions via Jacobi and Weierstrass elliptic functions.	Derives trigonometric, rational, hyperbolic, periodic, dark, kink, anti-kink, exponential forms, M-shaped solitons, and homoclinic breathers.
Dynamical analysis	Focuses on 2D and 3D graphical analysis of wave profiles to visualize morphological transitions under varying noise intensities.	Conducts dynamical analysis including phase portraits (bifurcation), time series for chaotic behavior, Lyapunov exponents, and sensitivity analysis.
Impact of noise	Identifies resilience thresholds: bright solitons are resilient to moderate noise ($\sigma \le 0.6$), but strong noise ($\sigma = 1.0$) destroys coherent structures.	Demonstrates the transition to chaos: increasing noise destabilizes solitons, and specific frequency adjustments drive the system from quasi-periodic motion to chaotic trajectories.

## Conclusion

In this work, the stochastic nonlinear Schrödinger equation with self-phase modulation in the dispersionless regime under multiplicative white noise was investigated. The improved modified extended tanh function method (IMETF) was used to derive exact analytical solutions for this stochastic nonlinear model.

The obtained solutions include bright and dark solitons, singular solutions, hyperbolic and trigonometric forms, as well as periodic and doubly periodic solutions expressed through Weierstrass elliptic functions. These solution families reflect the mathematical structure of the model and show how different wave patterns may arise in the presence of multiplicative white noise.

The analysis indicates that stochastic perturbations affect soliton dynamics in a manner that depends on the solution amplitude and the noise intensity. The presented plots show that, for moderate noise levels, the main features of the solutions are generally preserved, whereas stronger noise can noticeably alter their profiles. Differences between bright and dark soliton responses were also observed, with dark solitons appearing more sensitive to background fluctuations in the cases considered.

From a methodological point of view, the IMETF provides a structured way to construct exact solutions for the studied equation. The obtained results may be useful as analytical reference cases for future numerical and theoretical studies of stochastic nonlinear wave models.

Future work may consider higher-order effects, multidimensional extensions, and stability analysis under different types of stochastic perturbations. Such developments could help provide a broader understanding of stochastic wave propagation in nonlinear optical media^29–33^.

## Data Availability

This published article contains all of the data created or examined during this investigation
